# Computer-aided diagnosis system for predicting deeply invasive colorectal cancer: a comparison with a prospective study using magnifying chromoendoscopy

**DOI:** 10.1007/s00464-025-12289-w

**Published:** 2025-10-08

**Authors:** Tomoaki Matsumura, Mai Fujie, Yukihiro Nomura, Kenichiro Okimoto, Mamoru Tokunaga, Tsubasa Ishikawa, Naoki Akizue, Keisuke Matsusaka, Takuto Suzuki, Yoshiyasu Kitagawa, Kengo Nagashima, Toshiya Nakaguchi, Jun Kato

**Affiliations:** 1 Department of Gastroenterology, Graduate School of Medicine, Chiba University, Inohana 1-8-1, Chiba, 260-8670 Japan; 2 Department of Clinical Engineering Center, Chiba University Hospital, Chiba, Japan; 3 Center for Frontier Medical Engineering, Chiba University, Chiba, Japan; 4 Endoscopy Division, Chiba Cancer Center, Chiba, Japan; 5 Department of Diagnostic Pathology, Graduate School of Medicine, Chiba University, Chiba, Japan; 6 Biostatistics Unit, Clinical and Translational Research Center, Keio University Hospital, Tokyo, Japan

**Keywords:** Computer-aided diagnosis, Colorectal polyp, Colorectal cancer, Chromoendoscopy, Narrow-band imaging

## Abstract

**Background:**

Computer-aided diagnosis (CAD) systems for predicting deeply invasive colorectal cancer (CRC) have been developed, but their clinical utility remains uncertain. This study aimed to compare the diagnostic accuracy of a CAD system with that of endoscopists using magnifying narrow-band imaging (M-NBI) and magnifying chromoendoscopy (MCE).

**Methods:**

A total of 303 lesions obtained from a prospective multicenter study were analyzed. CAD diagnoses were classified as high- or low-confidence. The diagnostic accuracy of CAD was compared with that of M-NBI and MCE.

**Results:**

The diagnostic accuracy of the CAD system was 61.3%; sensitivity: 92.3%; specificity: 47.5%; positive predictive value (PPV): 95.4%; negative predictive value (NPV), and 89.1% overall accuracy. Among the 303 lesions, 193 (63.7%) were diagnosed with high confidence, with accuracy rates of 95.3% for high-confidence cases and 78.2% for low-confidence cases. For M-NBI, the sensitivity, specificity, PPV, NPV, and accuracy were 74.2%, 96.3%, 69.7%, 97.0%, and 94.1%, respectively. For MCE, the values were 83.9%, 95.2%, 66.7%, 98.1%, and 94.1%, respectively. The overall accuracy of the CAD system was lesser than that of M-NBI (*p* = 0.011) and MCE (*p* = 0.014). However, in high-confidence cases, the diagnostic performance of the CAD system was comparable to that of M-NBI and MCE.

**Conclusions:**

In high-confidence cases, the diagnostic accuracy rates of the CAD system were comparable to those of M-NBI and MCE. However, in low-confidence cases, the accuracy of the CAD system was insufficient, and further evaluations using M-NBI and MCE are necessary.

**Graphical abstract:**

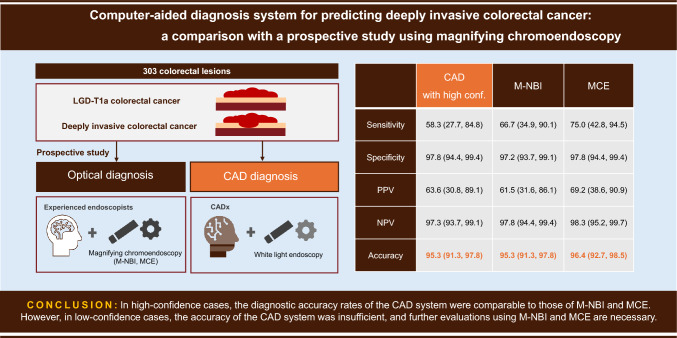

With the increasing prevalence of colorectal cancer (CRC) worldwide [1], the detection and excision of the important precursor lesions of CRC, which are the adenomas, is essential for cancer prevention [2]. Furthermore, CRC that has invaded the submucosa can be removed endoscopically, provided the invasion depth is shallow [3–5]. Therefore, distinguishing between CRC with invasion that is limited to the superficial submucosa and CRC with deep submucosal invasion, as well as between neoplastic and non-neoplastic lesions is absolutely necessary. The European Society of Gastrointestinal Endoscopy recommends the use of high-definition white light endoscopy in conjunction with (virtual) chromoendoscopy for predicting the presence and depth of any submucosal invasion in nonpedunculated colorectal polyps before initiation of any treatment [6]. However, the diagnosis of CRC with deep invasion is challenging in several cases [7–9], and misdiagnosis of deeply invasive CRC can lead to inappropriate treatment, and must be avoided.

In recent years, computer-aided detection/diagnosis (CAD) systems have been developed for supporting the optical diagnoses of colorectal polyps. There are two types of CAD systems, currently available for use with endoscopy: CADe, which focuses on detecting lesions, and CADx, which supports diagnosis. CADx has been reported to be useful for differentiating neoplastic lesions from non-neoplastic ones [10–12], distinguishing serrated lesions from hyperplastic polyps [13], and predicting the invasion depth of CRC [14–18]. CADx systems designed for predicting the invasion depth of CRC have been developed to be used in conjunction with ultra-magnifying endoscopy (endocytoscopy, 520 × magnification) [14], magnified NBI images (M-NBI) [15], and non-magnified white-light images (WLI) [16–18]. The reported diagnostic accuracy rates of CADx systems range from 87.3 to 94.1% [14–18]; however, these findings are based on preexisting still images used in retrospective studies. No prospective studies have yet been reported. The clinical utility of the CADx systems remains unclear, since optical diagnoses in clinical practice are not based on still images.

This study aimed to compare the diagnostic accuracy of the depths of invasion for CRC predicted by endoscopists in real time using magnifying chromoendoscopy with that predicted by a CADx system that analyzes WLI.

## Material and methods

### Study design

**Fig. 1:**
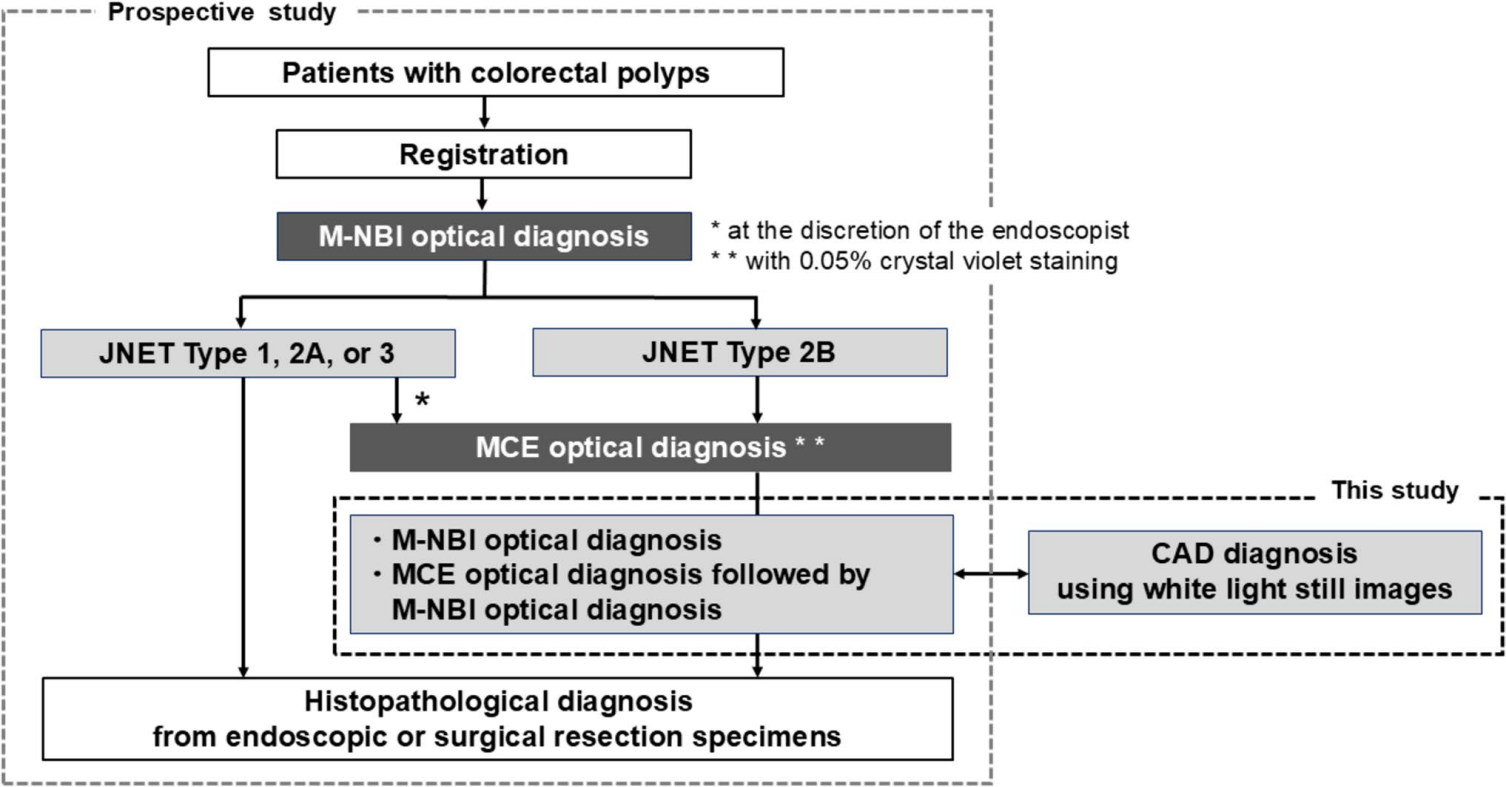
A flow diagram of the study. *M-NBI* narrow-band imaging magnified endoscopy, *MCE* magnified chromoendoscopy, *JNET* Japan NBI Expert Team

The study flow has been illustrated in Fig. 1. Among 1173 lesions registered in the multicenter prospective study (NBI-CV study) [19], 2374 images of 303 lesions examined with both M-NBI and magnifying chromoendoscopy (MCE) were analyzed from the 574 lesions registered at our hospital. The characteristics of the lesions have been summarized in Table 1. In this prospective study, the diagnostic accuracy of real-time, prospective diagnoses performed by experienced endoscopists using M-NBI and MCE was compared with that of a CADx system that analyzed all white-light still images obtained during endoscopic examinations. The endoscopists who participated in the study had previously performed more than one thousand colonoscopies and were highly experienced, possessing both diagnostic as well as therapeutic expertise with regards to colorectal polyps. They had routinely performed M-NBI and/or MCE examinations as well as endoscopic submucosal dissections.

**Table 1:** Characteristics of the lesions

	*n* = 303
Location, no. (%)	
Right colon	160 (52.8)
Left colon	81 (26.7)
Rectum	62 (20.5)
Macroscopic type, no. (%)	
Polypoid	131 (43.2)
Flat elevated	152 (50.2)
Depressed	20 (6.6)
Size, no. (%)	
≤ 5 mm	5 (1.7)
6–10 mm	39 (12.9)
11–20 mm	138 (45.5)
≥ 21 mm	121 (39.9)
Histopathology, no. (%)	
LGD	58 (19.1)
HGD	198 (65.3)
T1a CRC	16 (5.3)
T1b CRC	21 (6.9)
≥ T2 CRC	10 (3.3)

*LGD* low-grade dysplasia, *HGD* high-grade dysplasia, *T1a, T1* CRC with submucosal invasion depth < 1000 µm, *T1b, T1* CRC with submucosal invasion depth ≥ 1000 µm; ≥ T2: advanced CRC

### Patients and real-time optical diagnosi

Patients who were diagnosed with colorectal polyps and were scheduled for endoscopic or surgical resection were included in the study. Exclusion criteria included patients with clearly advanced CRC and those scheduled exclusively for surgical resection. Magnification-capable endoscopes (CF-H260AZI, PCF-Q260AZI, CF-HQ290I, CF-HQ290Z, or PCF-H290ZI; Olympus, Tokyo, Japan) were utilized for performing the colonoscopies. The gross morphology was assessed using white light imaging. Then, optical diagnosis by M-NBI observation was performed; M-NBI diagnosis was classified into four categories (JNET Types 1, 2A, 2B, and 3) according to the JNET classification [20], and the optical diagnosis at that time was registered. Diagnostic confidence (high or low) was also registered. After M-NBI observation, polyps were then stained with 0.05% CV (crystal violet) solution and MCE was performed on lesions judged by endoscopists as insufficient by M-NBI and those classified as Type 2B as per the JNET classification. After staining the polyps with a 0.05% crystal violet (CV) solution, MCE was performed on lesions deemed insufficiently evaluated by M-NBI or those classified as Type 2B in accordance with the JNET classification. According to the pit pattern classification, MCE diagnoses were categorized into eight groups (I, II, IIIs, IIIL, IV, VI low grade, VI high grade, and VN) [21]. Optical diagnosis by M-NBI and MCE was classified into four categories: hyperplastic polyp (HP)/sessile serrated adenoma/polyp (SSP), low-grade dysplasia (LGD), HGD-T1 CRC with submucosal invasion depth < 1000 µm (T1a), and T1 CRC with submucosal invasion depth ≥ 1000 µm (T1b)- advanced CRC (≥ T2). Afterward, endoscopic or surgical resection was performed, and a valid diagnosis was defined as the agreement between optical and postoperative histopathological diagnoses.

### CADx system

The CADx system used in this study used non-magnified white-light still images to diagnose the invasion depth of CRC by classifying it into two categories: LGD to T1a CRC, or T1b CRC and deeper [16]. When CAD diagnoses differed among images of the same lesion, the diagnosis with the highest frequency was chosen and was defined as a CAD diagnosis with low confidence. The lesion was classified as noninvasive cancer if the CAD diagnoses differed but occurred with equal frequency. Conversely, the diagnosis was defined as a CAD diagnosis with high confidence when all CAD diagnoses were consistent across the images.

### Histopathology

Histological analysis was performed in accordance with the Vienna classification [22]. The depth of the submucosal invasion was measured in accordance with the Japanese guidelines [23].

### Statistical analyses

Numbers and proportions were used to summarize the baseline characteristics. The primary aim of this study was to evaluate the diagnostic performance of CADx in comparison with M-NBI and MCE examinations for colorectal polyps. The McNemar test was used to compare the accuracy of CADx with that of M-NBI and MCE. For the optical diagnoses of CRC with deep invasion, sensitivity, specificity, positive predictive value (PPV), and negative predictive value (NPV) were calculated with exact binomial 95% confidence intervals (CIs) in addition to accuracy. The secondary objective of this study was to evaluate the results based on the diagnostic confidence of CADx. The IBM SPSS Statistics (version 29.0; IBM, Chicago, IL) and R software (version 4.4.2; R Foundation for Statistical Computing, Vienna, Austria) were used for performing the statistical analyses. A two-sided *p* value < 0.05 was considered statistically significant.

All authors had access to the study data and reviewed and approved the final manuscript.

### Ethics

The ethics committee at Chiba University reviewed and approved this study (approval number, M10295), and it was conducted in accordance with the Declaration of Helsinki. The patients’ data were coded for ensuring anonymity. Informed consent was obtained from all patients who participated in the prospective study. The content of this study, which examined the usefulness of CADx, was not included at the time of obtaining consent for the prospective study; however, it was subsequently approved by the Ethics Committee after re-evaluation. The participants in the study were provided with the option to opt out.

## Results

### Per-image and per-lesion analysis

Table 2 shows the sensitivity, specificity, PPV, NPV, and accuracy rate of the CADx system for per-image and per-lesion analyses. A total of 2374 images of 303 lesions were analyzed. With regards to the diagnostic accuracy of the CADx system in detecting CRC with deep invasion, the sensitivity, specificity, PPV, NPV, and accuracy rate for all images were 68.7%, 85.5%, 46.3%, 93.7%, and 82.9%, respectively. The corresponding values for each lesion were 61.3%, 92.3%, 47.5%, 95.4%, and 89.1%, respectively.

**Table 2:** Sensitivity, specificity, PPV, NPV, and accuracy rate of CAD diagnosis of CRC with deep invasion

	CAD for all images (*n* = 2374)	CAD for each lesion (*n* = 303)	CAD with high conf. (*n* = 193)	CAD with low conf. (*n* = 110)
Sensitivity, %, (95% CI)	68.7 (63.6, 73.4)	61.3 (42.2, 78.2)	58.3 (27.7, 84.8)	63.2 (38.4, 83.7)
Specificity, %, (95% CI)	85.5 (83.8, 87.0)	92.3 (88.4, 95.2)	97.8 (94.4, 99.4)	81.3 (71.8, 88.7)
PPV, %, (95% CI)	46.3 (42.1, 50.6)	47.5 (31.5, 63.7)	63.6 (30.8, 89.1)	41.4 (23.5, 61.1)
NPV, %, (95% CI)	93.7 (92.5, 94.8)	95.4 (92.2, 97.6)	97.3 (93.7, 99.1)	91.4 (83.0, 96.5)
Accuracy, %, (95% CI)	82.9 (81.3, 84.4)	89.1 (85.0, 92.4)	95.3 (91.3, 97.8)	78.2 (69.3, 85.5)

*CAD* computer-aided diagnosis, *CRC* colorectal cancer, *PPV* positive predictive value, *NPV* negative predictive value, *CI* confidence interval

### Results by CAD confidence level

Table 3 shows the comparison of CAD diagnosis and real-time optical diagnosis by endoscopists for CRC with deep invasion. Tables 4 and 5 show the same comparison for high-confidence and low-confidence CAD diagnoses, respectively. Among the 303 lesions, 193 (63.7%) were diagnosed with high confidence. The accuracy rates for high-confidence- and low-confidence CAD diagnoses were 95.3% and 78.2%, respectively. The sensitivity, specificity, PPV, NPV, and accuracy rate for M-NBI were 74.2%, 96.3%, 69.7%, 97.0%, and 94.1%, respectively, while for MCE, these values were 83.9%, 95.2%, 66.7%, 98.1%, and 94.1%, respectively. The accuracy rate of CAD diagnosis for each lesion was inferior to that of M-NBI (*p* = 0.011) and MCE (*p* = 0.014); however, the results for lesions with high confidence in CAD diagnosis were comparable to those obtained with M-NBI and MCE.

**Table 3:** Comparison of CAD diagnosis and real-time optical diagnosis by endoscopists for CRC with deep invasion

	CAD (*n* = 303)	M-NBI (*n* = 303)	MCE (*n* = 303)	*P* value (CAD vs M-NBI)	*P* value (CAD vs MCE)
Sensitivity, %, (95% CI)	61.3 (42.2, 78.2)	74.2 (55.4, 88.1)	83.9 (66.3, 94.5)	0.285	0.052
Specificity, %, (95% CI)	92.3 (88.4, 95.2)	96.3*(93.3, 98.2)	95.2 (92.0, 97.4)	0.016*	0.103
PPV, %, (95% CI)	47.5 (31.5, 63.7)	69.7 (51.3, 84.4)	66.7 (49.8, 80.9)	–	–
NPV, %, (95% CI)	95.4 (92.2, 97.6)	97.0 (94.2, 98.7)	98.1 (95.6, 99.4)	–	–
Accuracy, %, (95% CI)	89.1 (85.0, 92.4)	94.1*(90.8, 96.4)	94.1*(90.8, 96.4)	0.011*	0.014*

*CAD* computer-aided diagnosis, *M-NBI* narrow-band imaging magnified endoscopy, *MCE* magnified chromoendoscopy, *PPV* positive predictive value, *NPV* negative predictive value, *CI* confidence interval

*P < 0.05 indicated statistically significant differences

**Table 4:** Comparison of CAD diagnosis and real-time optical diagnosis by endoscopists for CRC with deep invasion: CAD diagnosis with high confidence

	CAD with high conf. (*n* = 193)	M-NBI (*n* = 193)	MCE (*n* = 193)	*P* value (CAD vs M-NBI)	*P* value (CAD vs MCE)
Sensitivity, %, (95% CI)	58.3 (27.7, 84.8)	66.7 (34.9, 90.1)	75.0 (42.8, 94.5)	0.655	0.414
Specificity, %, (95% CI)	97.8 (94.4, 99.4)	97.2 (93.7, 99.1)	97.8 (94.4, 99.4)	0.655	1.000
PPV, %, (95% CI)	63.6 (30.8, 89.1)	61.5 (31.6, 86.1)	69.2 (38.6, 90.9)	–	–
NPV, %, (95% CI)	97.3 (93.7, 99.1)	97.8 (94.4, 99.4)	98.3 (95.2, 99.7)	–	–
Accuracy, %, (95% CI)	95.3 (91.3, 97.8)	95.3 (91.3, 97.8)	96.4 (92.7, 98.5)	1.000	0.527

*CAD* computer-aided diagnosis, *M-NBI* narrow-band imaging magnified endoscopy, *MCE* magnified chromoendoscopy, *PPV* positive predictive value, *NPV* negative predictive value, *CI* confidence interval

**Table 5:** Comparison of CAD diagnosis and real-time optical diagnosis by endoscopists for CRC with deep invasion: CAD diagnosis with low-confidence

	CAD with low conf. (*n* = 110)	M-NBI (*n* = 110)	MCE (*n* = 110)	*P* value (CAD vs M-NBI)	*P* value (CAD vs MCE)
Sensitivity, %, (95% CI)	63.2 (38.4, 83.7)	78.9 (54.4, 93.9)	89.5 (66.9, 98.7)	0.317	0.059
Specificity, %, (95% CI)	81.3 (71.8, 88.7)	94.5 (87.6, 98.2)	90.1 (82.1, 95.4)	0.003*	0.074
PPV, %, (95% CI)	41.4 (23.5, 61.1)	75.0 (50.9, 91.3)	65.4 (44.3, 82.8)	–	–
NPV, %, (95% CI)	91.4 (83.0, 96.5)	95.6 (89.0, 98.8)	97.6 (91.7, 99.7)	–	–
Accuracy, %, (95% CI)	78.2 (69.3, 85.5)	91.8*(85.0, 96.2)	90.0*(82.8, 94.9)	0.003*	0.012*

*CAD* computer-aided diagnosis, *M-NBI* narrow-band imaging magnified endoscopy, *MCE* magnified chromoendoscopy, *PPV* positive predictive value, *NPV* negative predictive value, *CI* confidence interval

*P < 0.05 indicated statistically significant differences

### Results by gross morphology

**Fig. 2:**
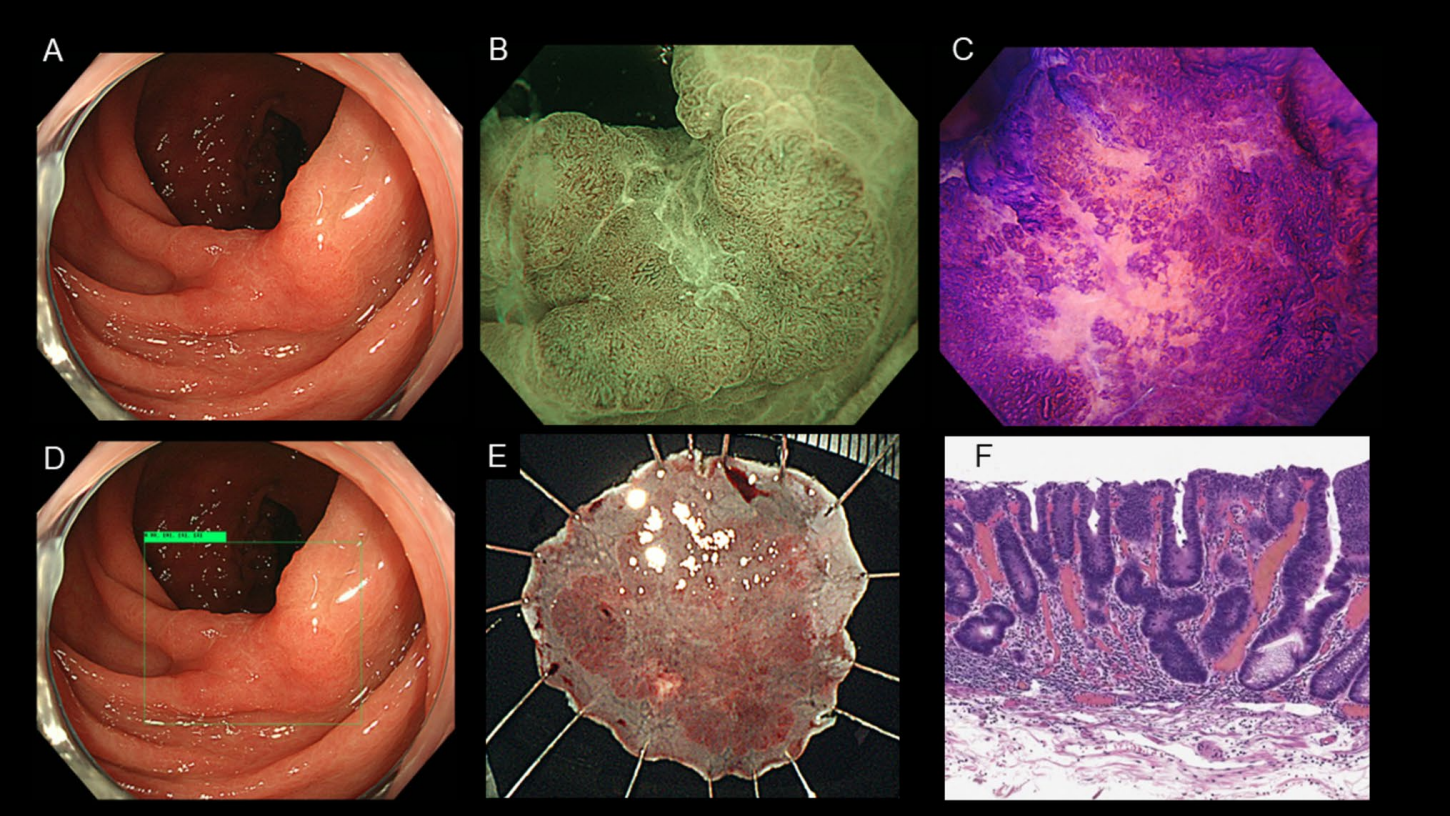
A case in which CAD was useful for diagnosis. A 15-mm lesion in the sigmoid colon. **A** White light imaging. **B** M-NBI imaging: The central area of the depression was covered with mucus, preventing observation of the vascular and mucosal structures. The visible surface pattern was irregular, and the lesion was classified as JNET Type 2B. **C** MCE imaging: A distorted pit within a well-demarcated area was observed and classified as pit pattern VI high grade (invasive pattern). The optical diagnosis indicated T1b CRC. **D** CAD diagnosis: This lesion was identified as a non-deeply invasive cancer (LGD-T1a CRC) with high confidence. **E** Specimen resected via endoscopic submucosal dissection. **F** Final histopathologic diagnosis: HGD. *CAD* computer-aided diagnosis, *CRC* colorectal cancer, *M-NBI* narrow-band imaging magnified endoscopy, *MCE* magnified chromoendoscopy, *HGD* high-grade dysplasia, *T1b, T1* colorectal cancer with submucosal invasion depth ≥ 1000 µm

The accuracy rates for polypoid, flat elevated, and depressed lesions for CAD were 84.7%, 92.8%, and 90.0%, for M-NBI, they were 88.5%, 99.3%, and 90.0%, and for MCE, they were 89.3%, 99.3%, and 85.0%, respectively. M-NBI and MCE diagnoses were significantly superior to CAD diagnoses for flat elevated lesions. In contrast, for depressed lesions, the diagnostic accuracy of CAD was comparable to that of M-NBI and higher than that of MCE, although no statistical difference was detected. Figure 2 depicts cases in which CAD was useful for the diagnosis of depressed lesions.

### Discussion

The optical diagnosis of CRC with deep invasion is critical in determining the subsequent treatment. This is the first report to examine the usefulness of CADx for the prediction of the depth of CRC in actual clinical practice. CADx used white light non-magnified images, and its diagnostic accuracy for high-confidence diagnoses was comparable to the accuracy of experienced endoscopists using magnifying endoscopy. However, with regards to CAD diagnoses with low-confidence, the accuracy was significantly inferior to that of M-NBI and MCE diagnoses, indicating the requirement of magnifying chromoendoscopy in such cases.

Three types of CADx systems have been reported for diagnosing CRC with deep invasion. Tamai et al. developed a CADx system using M-NBI that had a sensitivity of 83.9% and a specificity of 82.6% [15]. The diagnostic accuracy of a CADx system using endocytoscopy (520 × magnifying endoscope) developed by Takeda et al. was reportedly 94.1% [14]. The authors of this study created a more practical CADx system that used WLI for diagnoses and demonstrated a diagnostic accuracy of 90.3% [16]. Nemoto and Luo et al. also developed a CADx based on WLI and reported its usefulness [17, 18]. However, all the reports mentioned above have been retrospective studies that have been performed using still images taken in the past, and their applicability in actual clinical practice remained unknown. In actual clinical practice, diagnoses are made during endoscopic examinations using moving images and are not based on still images. Furthermore, various guidelines recommend the use of image-enhanced endoscopy (IEE), such as NBI which is widely used in conjunction with magnifying endoscopes [3, 4, 6]. Therefore, it is essential to evaluate the practical usefulness of CADx by comparing it with chromoendoscopy using magnifying endoscopes. Previously conducted multicenter prospective studies have reported that the optical diagnostic accuracy of CRC with deep invasion using chromoendoscopy with magnifying endoscopy was between 71.2% and 98.8% [9, 19, 24, 25]. We believe that the results of this study demonstrate the clinical usefulness of CADx, considering the absence of prospective studies on CADx. On the other hand, in recent years, the usefulness of CADx in differentiating between neoplasms and non-neoplasms has been evaluated by some prospective studies [11, 12, 26–28]. Rex et al. compared the sensitivity and specificity of CADx-assisted histological predictions for colorectal polyps ≤ 5 mm with the diagnoses of general endoscopists. This study indicated that the sensitivity of the CADx-assisted histological predictions and those of the general endoscopists was similar (90.7% vs. 90.8%; *p* = 0.52), however, the specificity of the CADx-assisted group though higher, was insufficient (59.5% vs. 64.7%; *p* < 0.001) [11]. A prospective multicenter study conducted by Li et al. compared the accuracy of CADx in diagnosing adenomatous polyps with that of experienced endoscopists. They reported that the accuracy of CADx was 71.6% (95% CI: 68.0–75.0), which was significantly inferior compared with the accuracy of experienced endoscopists (75.2% [95% CI 71.7–78.4]; *p* = 0.023) [12]. The current study also demonstrated that the overall accuracy of CADx was significantly lower than that of endoscopists. The results of this study indicate that the usefulness of CADx may not be sufficient. A previous retrospective study conducted by us demonstrated the usefulness of CADx using the same CADx system [16]; however, according to the current study, the usefulness of CADx was lower than anticipated. One possible reason for this difference could be attributed to the fact that while the previous study only used white light and still images, in this study, the diagnoses of CADx were compared to diagnoses made by experienced endoscopists using magnifying endoscopes. During endoscopic examinations, moving images enable the observation of dynamic changes (such as the mobility and hardness of tumors), which are difficult to capture in still images. In addition, the results were possibly affected by factors including detailed examinations using magnifying endoscopes, NBI, and pit pattern classification. In this study, an attempt was made to improve the diagnostic accuracy of the CADx system [16] by adding training data, which included 1304 images of 231 HGD lesions and 201 images of 33 T1b CRC lesions, and by replacing the base model with EfficientNet-B4 [29]; however these have not been detailed in this report. However, the diagnostic accuracy of CADx was not significantly positively influenced, indicating that further improvement in the diagnostic accuracy of white light non-magnified images might be challenging. However, when stratified by lesion morphology, the diagnostic accuracy of CADx for depressed lesions was comparable to that of M-NBI and higher than that of MCE, although no statistical difference was detected. In particular, depressed-type lesions such as non-granular laterally spreading tumors with pseudo-depression have been reported to be more difficult to assess accurately compared with protruded or flat-type lesions, even when using advanced endoscopic techniques such as M-NBI and MCE [30]. This difficulty is thought to be associated with factors such as the presence of fibrosis even in non-invasive lesions, as well as multifocal patterns of submucosal invasion, both of which may lower diagnostic accuracy. Our CADx system may have contributed to improved diagnostic performance for depressed lesions by evaluating the overall lesion morphology based on a large number of learned images, rather than relying solely on subtle microstructural features.

The strength of this study lies in highlighting the usefulness of CADx for diagnosing invasive CRC by comparing its diagnostic accuracy with the real-time diagnostic accuracy of endoscopists in actual clinical practice. The accuracy of CADx diagnoses with high confidence was the same as that of experienced endoscopists using magnifying endoscopes, and did not require additional testing using magnification. Unlike CADe, which supports detection, CADx, which supports diagnosis, does not allow endoscopists to easily reject CAD diagnoses in real-time. In other words, experience is required to decide whether to accept or reject the CAD diagnosis on the spot. Although this has not been investigated in the current study, CADx diagnoses with high confidence could be useful for trainee endoscopists.

This study has several limitations. First, CAD was not used for real-time, prospective diagnoses. However, since CAD diagnoses are based on still images, we believe that similar results would be obtained even if CAD was used in real time. Second, the comparison was made with endoscopic specialists, not trainee endoscopists. Previous reports on CAD have demonstrated that CAD diagnoses are superior to those of trainee endoscopists and comparable to those of experienced endoscopists. As in our previous study [16], CAD could have been adjudged as superior when compared with trainee endoscopists. Third, the additive effect of CAD on endoscopists was not investigated. Further research is needed to explore the impact of CAD diagnoses on the performance of endoscopists.

In conclusion, the diagnostic accuracy of CADx for diagnosing CRC with deep invasion was compared with the diagnostic accuracy of endoscopists in this study. In the future, further improvements are required in the performance of CADx to make it more useful in clinical practice for diagnosing CRC with deep invasion.
